# Reflectance Spectroscopy with Multivariate Methods for Non-Destructive Discrimination of Edible Oil Adulteration

**DOI:** 10.3390/bios11120492

**Published:** 2021-12-02

**Authors:** Ning Su, Shizhuang Weng, Liusan Wang, Taosheng Xu

**Affiliations:** 1 Institute of Intelligent Machines, Hefei Institutes of Physical Science, Chinese Academy of Sciences, Hefei 230031, China; oksuning@mail.ustc.edu.cn; 2 Intelligent Agriculture Engineering Laboratory of Anhui Province, Hefei 230031, China; 3 National Engineering Research Center for Agro-Ecological Big Data Analysis and Application, Anhui University, Hefei 230601, China; weng_1989@126.com

**Keywords:** oil adulteration, Vis-NIR, reflectance spectroscopy, multivariate analysis, CARS

## Abstract

The visible and near-infrared (Vis-NIR) reflectance spectroscopy was utilized for the rapid and nondestructive discrimination of edible oil adulteration. In total, 110 samples of sesame oil and rapeseed oil adulterated with soybean oil in different levels were produced to obtain the reflectance spectra of 350–2500 nm. A set of multivariant methods was applied to identify adulteration types and adulteration rates. In the qualitative analysis of adulteration type, the support vector machine (SVM) method yielded high overall accuracy with multiple spectra pretreatments. In the quantitative analysis of adulteration rate, the random forest (RF) combined with multivariate scattering correction (MSC) achieved the highest identification accuracy of adulteration rate with the full wavelengths of Vis-NIR spectra. The effective wavelengths of the Vis-NIR spectra were screened to improve the robustness of the multivariant methods. The analysis results suggested that the competitive adaptive reweighted sampling (CARS) was helpful for removing the redundant information from the spectral data and improving the prediction accuracy. The PLSR + MSC + CARS model achieved the best prediction performance in the two adulteration cases of sesame oil and rapeseed oil. The coefficient of determination ($R_{P_{cv}}^{2}$) and the root mean square error ($RMSE_{P_{cv}}$) of the prediction set were 0.99656 and 0.01832 in sesame oil adulterated with soybean oil, and the $R_{P_{cv}}^{2}$ and $RMSE_{P_{cv}}$ were 0.99675 and 0.01685 in rapeseed oil adulterated with soybean oil, respectively. The Vis-NIR reflectance spectroscopy with the assistance of multivariant analysis can effectively discriminate the different adulteration rates of edible oils.

## 1. Introduction

Edible oils are rich in essential nutrients for human beings and thus are some of the most common cooking ingredients in our daily lives [1]. However, different kinds of oils have different compositions and contents of substances that are beneficial to human health. For example, rapeseed oil contains more tocopherols [2], and sesame oil is rich in linoleic acid, oleic acid, and linolenic acid [3]. Fish oil is rich in essential fatty acids, particularly the omega-3 polyunsaturated fatty acids (n-3 PUFA) [4]. Because the price of edible oil is highly dependent on its nutritional content, some high-value-added oils are frequently subjected to adulteration with low-quality oils in the production process to obtain undue commercial profits [3,5]. In addition, in some cases, some edible oils are even contaminated by gutter oil from kitchen waste or industrial oil [6]. The unqualified oil not only harms the economic interests of consumers but also poses an invisible threat to human health [7,8]. The adulteration of edible oil is prone to occur in the production process and has become a serious worldwide public health problem [9]. Ensuring the quality and safety of edible oil is a major challenge in food inspection. Hence, it is essential to develop effective and convenient methods to identify the adulteration of edible oils.

Although edible oils have obvious characteristics, such as odor and color, which can be used in preliminary artificial discriminations, the judgments are too subjective and susceptiblebased on these natural characteristics. Some researchers developed sensors to accurately sense the external characteristics of oils. For example, the electronic tongue was developed to qualitatively detect adulteration based on tasting fingerprints [10,11]. However, the mechanical structure of the sensor is complex, and passivation occurs after long-term use. In addition to the sensor problems, the discrimination methods of oil quality are divided into two main research directions. In the first stream, chemical methods, such as gas chromatography (GC) [12] and inductively coupled plasma mass spectrometry (ICP-MS) [13], usually determine the quality of oil through specific trace component detection. The GC and ICP-MS have been successfully applied to detect polycyclic aromatic hydrocarbons, phthalate esters, and alkylphenols in edible vegetable oils [14] and trace elements (e.g., Cu, Ge, Mn etc.) [15]. In addition, chemical methods are standard tests with high accuracy and sensitivity. However, they always require complex pretreatments and professional operations and are not suitable for quick discrimination. The development of optical detection technology has led to the second stream of research. Researchers use spectroscopic methods such as near-infrared spectroscopy (NIR) to identify different oil types. The NIR can obtain molecular vibration and rotation information by collecting the absorption spectrum of the target. It is a nondestructive testing method that does not consume chemical reagents. In view of these advantages, the NIR has been used to identify adulterated *Camellia oleifera* [16], identify hogwash oil in blended oil, and classify nontransgenic and transgenic oil samples [17]. Nevertheless, the NIR spectrum is limited to the near-infrared band and lacks some key features of other wavebands [18].

The visible and near-infrared (Vis-NIR) reflectance spectrum is generated by the absorption of radiation, which causes the molecular vibration from the ground state to a higher internal energy level. Therefore, it reflects the internal and external features of the objects. In previous studies, the Vis-NIR reflectance spectroscopy has been used to develop a hybrid proximal sensing method to quickly identify oil-contaminated soil [19] and measure the spectral characteristics of winter wheat canopy [20]. The Vis-NIR reflectance spectroscopy can obtain abundant feature information yet requires simple pretreatment and easy operation and thus exhibits great potential for the rapid identification of adulterated oils. However, olive oil is the main research object for adulteration identification in previous studies. Studies on the adulteration of sesame oil, soybean oil, and rapeseed oil widely consumed in developing countries are still lacking. A systematic solution has not yet been formed because of the lack of multivariate data preprocessing and modeling methods for edible oil adulteration research.

**Figure 1:**
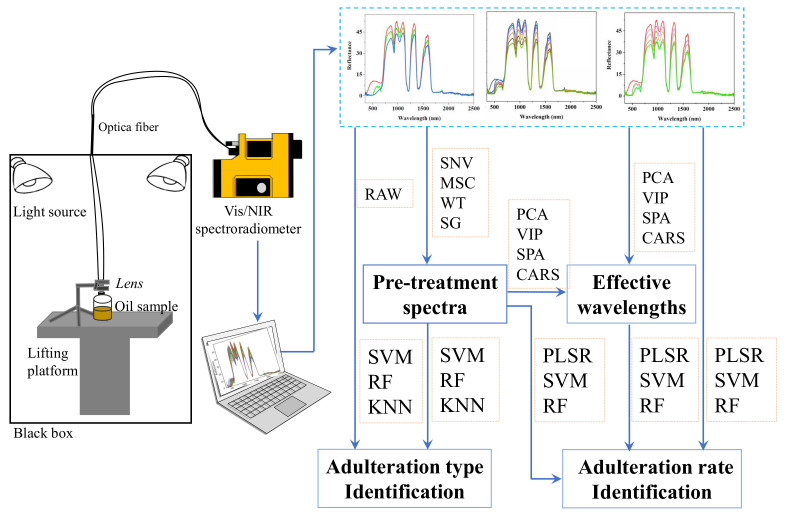
Schematic of measurement of Vis-NIR reflectance spectroscopy.

In this study, we presented a nondestructive and effective method for identifying the adulteration of edible oil using Vis-NIR reflectance spectroscopy. The experimental workflow of oil adulteration detection is shown in Figure 1. A spectroradiometer system was established to collect the Vis-NIR reflectance spectra of adulterated oil samples. The three most commonly consumed edible oils (i.e., soybean oil, sesame oil, and rapeseed oil) were selected as the analysis objects. Multivariant analysis methods, such as partial least squares regression (PLSR) [21], support vector machine (SVM) [22], random forest (RF) [23], and K-nearest neighbor (KNN) [24] were applied for qualitatively determining the adulteration types in oil samples. Beyond the qualitative analysis, the partial least squares regression (PLSR), support vector regression (SVR) [25], and random forest (RF) were used for accurate quantitative analysis to determine the adulteration rates of oil samples. Four pretreatment methods, including standard normal variables (SNV), multivariate scattering correction (MSC), Savitzky-Golay (SG) smoothing, and wavelet transform (WT) were used to process the raw Vis-NIR spectra data. In order to establish an efficient and simplified practical model, the outcomes of four effective wavelength selection algorithms, such as principal component analysis (PCA), variable importance in projection (VIP), successive projections algorithm (SPA), and competitive adaptive reweighted sampling (CARS), were compared and evaluated in this paper.

## 2. Materials and Methods

### 2.1. Preparation of Experimental Samples

In order to obtain pure samples of rapeseed oil, sesame oil, and soybean oil, the rapeseed seed (Yangguang 198), sesame seed (Xinyuzhi 10), and soybean (Wandou 33) were processed with an oil press. The soybean oil was taken as the adulterated oil to blend with sesame oil and rapeseed oil, respectively. The doping ratios were set as 0%, 10%, 20%, 30%, 40%, 50%, 60%, 70%, 80%, 90%, and 100%. The 0% represented the pure soybean oil, and the 100% represented the pure sesame oil or rapeseed oil. In our experiment, five groups of adulterated samples were generated to ensure the independence of the oil samples. Each group included nine adulterated sesame oils, nine adulterated rapeseed oils, and four pure oils (one sesame oil, one rapeseed oil and two soybean oils for different adulteration experiments). Therefore, there were a total of 110 oil samples for the adulteration experiments of sesame oil and rapeseed oil.

### 2.2. The Measurement of Reflectance Spectra

In order to obtain the Vis-NIR reflectance spectra, a simple spectrum measurement platform was constructed. The platform was mainly composed by a black box, four halogen lamps (75w), an automatic lifting sample system, a PSR-3500 portable spectroradiometer, spectrum acquisition software, and so on, as shown in Figure 1. The portable spectroradiometer can continuously acquire reflection spectrum of objects in the range of 350–2500 nm with 1 nm resolution. The spectral acquisition experiments were conducted in the black box to avoid the interference of external light. The oil samples were placed on the automatic lifting platform with a PVC white board. The height of the lens from the top surface of oil sample was adjusted to 3 cm. The reflectance spectra of the empty glass beaker and white board were measured before spectral acquisition experiments of the oil samples to calibrate the spectrometer [26]. Finally, a total of 110 Vis-NIR reflectance spectra were obtained for multivariate analysis.

### 2.3. Preprocessing of Reflectance Spectra

The SNV, MSC, SG smoothing, and WT are commonly used to remove the negative effect of various spectra data [27]. The SNV method is used to correct the spectral difference caused by particle scattering among different experimental samples. Similar to the SNV, MSC is a technique for eliminating the influence of scattering caused by the particle size, density, and uniformity of the distribution of samples [28,29]. SG smoothing adopts a fix-size time domain window moving full wavelength to smooth the noise as much as possible without compromising the original spectrum [30]. WT is a method for local signal analysis in both time domain and frequency domain. It can locate useful information in huge signal data and conduct accurate time-domain and frequency-domain analysis [31]. In addition, the origin spectra were also applied for a comparative analysis. In this paper, we conducted a systematic comparison to evaluate the effectiveness of the four pretreatment methods in the application of Vis-NIR reflectance spectroscopy.

### 2.4. Selection of Effective Wavelengths of Reflectance Spectra

In order to eliminate redundant information and simplify the prediction models, the PCA, VIP, SPA, and CARS were adopted to select the effective wavelengths [32]. PCA is a classical method for dimension reduction. The top loading vectors in PCA represent the importance of different features. The wavelengths with larger absolute values in loading vectors possess most of the characteristic information of the original spectra. VIP is a variable selection method for ranking different individual wavelengths. It evaluates the correlation between each individual wavelength and its response variable using a cutoff criterion to segment relevant/irrelevant variables [33]. SPA measures the importance of each variable by comparing the normal prediction error of the same variable with the distribution of the displacement prediction error [34]. CARS uses the adaptive reweighted sampling technique to select the wavelengths with larger absolute value of regression coefficient in a partial least square model [35]. Then, a 10-fold cross-validation was used to select a subset of wavelengths with the lowest root mean square error as the optimal feature set.

### 2.5. Adulteration Types and Adulteration Rate Prediction Models

To identify the different adulteration types of oil samples, the SVM, RF, and KNN methods were employed to build the classification models, respectively. SVM maps spectral data into high-dimensional space by using kernel function and seeks for the optimal hyperplane to separate samples. RF is a classifier with multiple-decision trees to classify samples and assess the importance of variables. KNN is a classifier insensitive to outliers that can classify by measuring the distance between different feature values. KNN predicts new sample according to its K-nearest neighbors.

In addition, three regression models including PLSR, SVR, and RF were adopted to predict the oil adulteration rate, which was a very important quantitative indicator for oil quality inspection. PLSR is a commonly used multivariate analysis method in the Vis-NIR spectrum analysis, which constructs a regression model of multiple dependent variables against multiple independent variables. SVR was developed based on the structural risk minimization principle. It is suitable for the analysis of high-dimensional spectral data with a small sample set. RF can be used for regression modeling, and the predicted value in RF regression model is the average output of each decision tree. RF can reduce the risk of overfitting by the process of averaging decision trees. Therefore, it is commonly applied for the modeling and analysis of various spectral data.

### 2.6. Performance Evaluation

In the analysis of the adulteration type, the performance of prediction model was evaluated by accuracy (ACC), defined as Equation (1). The closer ACC is to 1, the better performance the evaluated model is. In this paper, the ACC of the calibration set is represented as $ACC_{C}$, and the ACC of the prediction set is represented as $ACC_{P}$.

(1) $$ACC=\frac{m_{k}}{n}$$

where $m_{k}$ is the number of correctly predicted samples and *n* is the total number of samples.

For the quantitative analysis of adulteration rate, the performance of the regression model was evaluated by the coefficient of determination ($R^{2}$) and root mean square error (RMSE). In the calibration set, the $R^{2}$ and RMSE were represented as $R_{C}^{2}$ and $RMSE_{C}$, respectively, and $R_{P}^{2}$ and $RMSE_{P}$ for the prediction set. The value of $R^{2}$ is between 0 and 1, reflecting the relative degree of regression contribution. The closer $R^{2}$ is to 1, the better the model is. RMSE evaluates the deviation of the predicted value from the measured value. A smaller RMSE represents a better model performance. The formulas of the $R^{2}$ and RMSE are shown in Equations (2) and (3).

(2) $$\begin{array}{cc} R^{2} & =1-\frac{\sum_{i=1}^{n}{\left(y_{i}-{\hat{y}}_{i}\right)}^{2}}{\sum_{i=1}^{n}{\left(y_{i}-\bar{y}\right)}^{2}} \end{array}$$

(3) $$\begin{array}{cc} RMSE & =\sqrt{\frac{\sum_{i=1}^{n}{\left(y_{i}-{\hat{y}}_{i}\right)}^{2}}{n}} \end{array}$$

where *n* is the number of the samples, $y_{i}$ is the measured value of the ith sample, ${\hat{y}}_{i}$ is the predicted value of the ith sample, and $\bar{y}$ is the average value of all the $y_{i}$.

**Figure 2:**
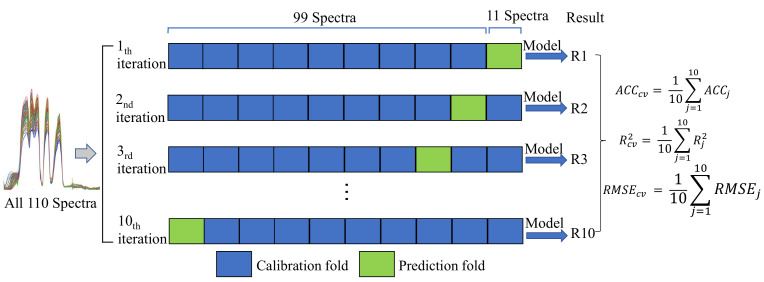
10-fold crossvalidation of the Vis-NIR spectra analysis.

Furthermore, a 10-fold cross validation was applied to evaluate the robustness and generalization of predictive models. In this paper, the spectral data were randomly divided into ten partitions with equal size. Nine of the ten partitions were combined as the training data (calibration set), and the remaining one partition was used as validation data (prediction set). The crossvalidation procedure was repeated 10 times with each of the 10 partitions used exactly once as the validation data. The 10-fold crossvalidation of the Vis-NIR spectra analysis was shown in Figure 2. The final results were the average of the 10 times estimation.

## 3. Results and Discussion

### 3.1. The Vis-NIR Reflectance Spectra of the Adulteration Oil Samples

**Figure 3:**
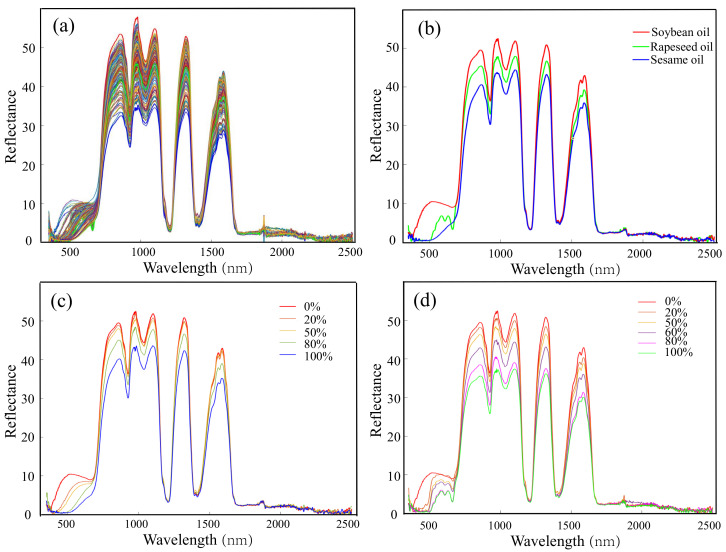
The Vis-NIR reflectance spectra of the adulterated oils and pure oils. (**a**) The overview of all the spectra; (**b**) spectra of pure sesame oil, rapeseed oil, and soybean oil; (**c**) spectra of sesame oil adulterated with soybean oil in different ratios; and (**d**) spectra of rapeseed oil adulterated with soybean oil in different ratios.

The Vis-NIR reflectance spectra of the 110 adulterated oil samples were shown in Figure 3a. First, the overview of all the spectra highlighted two main aspects. The reflectivity in visible band is relatively lower than that of the most near-infrared band (780–1700 nm). Moreover, the reflectivity performed significant differences in partial visible band (380–700 nm). By contrast, the significant differences of the spectra mainly concentrated on some peaks in whole near-infrared band. The reflectance spectra of all oil samples coincided in the range of 1700–2500 nm with no special peaks or troughs. To investigate the inherent spectral characteristics of different oils, the spectra of the three pure oils were extracted and shown in Figure 3b. The spectra were similar in fluctuation trends but with different amplitudes in the Vis-NIR band, which was the critical characteristic that helped to inspect the composition of different oils. The largest amplitude difference located in visible band between 400 and 700 nm. The spectrum of sesame oil increased slowly, while the spectrum of soybean oil had a broad absorption peak, and the spectrum of rapeseed oil had two relatively narrow absorption peaks in this visible range. The reflectance spectra of sesame oils in different adulteration rates were shown in Figure 3c. The reflectivity was the main difference for the spectra in the range of 720–1700 nm. The reflectivity increased as the soybean oil doping ratio increased. Figure 3d presented the spectra of rapeseed oil adulterated with soybean oil in different proportions. With the increase in the adulteration rate of soybean oil, the reflectivity increased, and the spectral shape approached that of soybean oil. These spectral features preliminarily demonstrated that the Vis-NIR reflectance spectroscopy is feasible for oil adulteration.

### 3.2. Qualitative Analysis of Vis-NIR Spectra to Identify Oil Adulteration Type

In our study, the experimental samples were divided into seven classes, i.e., pure sesame oil (100%), low-adulterated sesame oil (60% ≤ ratio ≤ 90%), high-adulterated sesame oil (10% ≤ ratio ≤ 40%), pure soybean oil (0%), high-adulterated rapeseed oil (10% ≤ ratio ≤ 40%), low-adulterated rapeseed oil (60% ≤ ratio ≤ 90%), and pure rapeseed oil (100%). Due to the adulteration rate of 50% being rare in practice, the samples with adulteration rate of 50% were excluded from the experiment of qualitative analysis. In order to eliminate the external interference and noise, four pretreatment methods were used to preprocess the spectral data. The SVM, RF, and KNN were adopted to construct the prediction models for identifying an oil adulteration type based on the spectral data. The 10-fold crossvalidation was conducted to the each of the models, and the average prediction results were shown in Table 1. The SVM and RF achieved the accuracy of 100% with all the pretreatment methods in the calibration set. To be specific, the SVM model with SNV and MSC pretreatment methods also achieved the average accuracy of 100% in the prediction set. Compared with SVM and RF, KNN failed to obtain desirable prediction results in both the calibration set and prediction set. The poor accuracy in the prediction set further indicated that KNN method was apt to overfit in the Vis-NIR reflectance spectrum. Therefore, the KNN method was not recommended to identify the oil adulteration types. On the whole, the SVM method outperformed the other methods. Of note, the SNV and MSC pretreatment methods had the best performance in each of the three classification methods. Thus, the SNV and MSC pretreatments were recommended to identify oil adulteration types.

To further evaluate the performance of the model (SVM + SNV/MSC) in independent samples, six pure oils including two brands of sesame oil, two brands of rapeseed oil, and two brands of soybean oil were collected from the local Walmart stores for experiments. The Vis-NIR reflectance spectra were measured on the platform as described in Section 2.2. The constructed model (SVM + SNV) was chosen for the qualitative analysis of the six samples. All six samples were correctly classified to their categories based on the SVM + SNV model. The experimental results demonstrated that the presented model had good robustness for independent samples. Compared with the chromatography method [36], the Vis-NIR reflectance spectroscopy is a fast and nondestructive method for detecting oil adulteration with 100% identification accuracy, while the chromatography method relies on a series of chemical steps and skilled analytical technicians.

### 3.3. Quantitative Analysis with Full Wavelengths of Vis-NIR Spectra

The reflectance spectra were used to construct the PLSR, SVR, and RF regression models to determine the accurate adulteration rates of sesame oil and rapeseed oil. For accurate quantitative analysis, the adulteration rates of sesame oil and rapeseed oil were analyzed separately in our experiments. Similar to the identification of the adulteration type, the four pretreatment methods and raw spectra were applied to compare the prediction accuracy of the adulteration rates. The prediction result of each model was the average prediction results of 10-fold crossvalidation.

**Figure 4:**
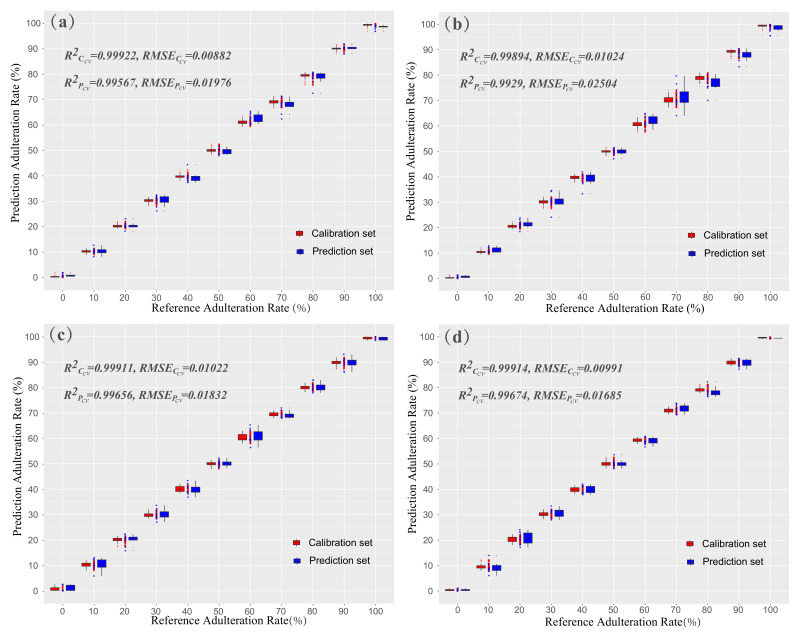
Prediction results of full and effective wavelengths. (**a**) The predation results of sesame oil adulterated with soybean oil using RF and MSC pretreatment in the calibration set and prediction set; (**b**) the predation results of rapeseed oil adulterated with soybean oil using RF and MSC pretreatment in the calibration set and prediction set; (**c**) the prediction results of sesame oil adulterated with soybean oil using the model of PLSR + MSC + CARS in the calibration set and prediction set; and (**d**) the prediction results of rapeseed oil adulterated with soybean oil using the model of PLSR + MSC + CARS in the calibration set and prediction set.

In the study of sesame oil adulterated with soybean oil, the Vis-NIR spectra were analyzed by the PLSR, SVR, and RF methods combined with different pretreatments. Table 2 showed the average prediction results based on 10-fold crossvalidation. The optimal result in PLSR method was obtained with WT pretreatment ($R_{P_{cv}}^{2}$ = 0.97282, $RMSE_{P_{cv}}$ = 0.05116). The RF model achieved its optimal performance using the MSC pretreatment ($R_{P_{cv}}^{2}$ = 0.99567, $RMSE_{P_{cv}}$ = 0.01976). While the SVR achieved its optimal performance using the MSC pretreatment ($R_{P_{cv}}^{2}$ = 0.98209, $RMSE_{P_{cv}}$ = 0.04083). All three regression methods with different pretreatments can achieve high prediction performance. The results proved that the Vis-NIR spectra had enormous potential for discriminating the adulteration of oils with high robustness in different prediction models. Overall, the RF model with the MSC pretreatment had the best performance in predicting sesame oil adulterated with soybean oil. The detailed results of the RF model with the MSC pretreatment are shown in Figure 4a. The red points represent the samples in calibration set, and blue points represent the samples in prediction set. The dispersion of the blue points in each adulteration rate was larger than the corresponding red points. The boxplots in two sides (red boxplots and blue boxplots) of each adulteration rate indicated the statistical characteristics of the prediction results. The horizontal lines at the bottom and roof of the boxplots represented the values of lower quartile and upper quartile of the data distribution, respectively. The horizontal lines in the middle of the boxplots were the median value of the data distribution. There were some data points beyond the overall boxplot being regarded as the outliers in the experiments.

In the study of rapeseed oil adulterated with soybean oil, we adopted the same analysis procedure as the sesame oil adulteration case. The analysis results were shown in Table 3. The PLSR method achieved its optimal prediction accuracy using SNV pretreatment ($R_{P_{cv}}^{2}$ = 0.99019, $RMSE_{P_{cv}}$ = 0.02902). The SVR method achieved its optimal performance using the SNV pretreatment ($R_{P_{cv}}^{2}$ = 0.9882, $RMSE_{P_{cv}}$ = 0.03281). The RF method combined with the MSC pretreatment achieved the best prediction performance among the three methods ($R_{P_{cv}}^{2}$ = 0.9929, $RMSE_{P_{cv}}$ = 0.02504), which was consistent with the result of the sesame oil adulteration case. It demonstrated that the RF method combined with MSC pretreatment was a robust model for identifying adulteration rates of sesame oil and rapeseed oil. The corresponding prediction results of the calibration set and prediction set were shown in Figure 4b. On the whole, the experiment results proved that the Vis-NIR reflectance spectroscopy can achieve accurate prediction and provided a new method for the quantitative detection of oil adulteration rates. In addition, the high-accuracy prediction results were mainly located at both ends (adulterationrate≤0.2 or adulterationrate≥0.8), which demonstrated that the Vis-NIR reflectance spectroscopy was more sensitive to pure edible oil. The identification ability of pure oil is exactly in line with the main demands of pure oil identification in practice.

### 3.4. Quantitative Analysis with Effective Wavelengths of Vis-NIR Spectra

In this section, we intend to evaluate whether effective wavelengths could help to promote the prediction accuracy of adulteration rates. The full wavelengths of the Vis-NIR reflectance spectra inevitably comprised the irrelevant features. Extracting optimal wavelengths can remove the influence of noncritical factors and reduce the complexity of prediction models. Furthermore, a simplified model can contribute to the downstream analysis of oil quality detection. Therefore, four effective spectral selection algorithms (i.e., PCA, SPA, VIP, and CARS) were applied to extract the effective wavelengths. The obtained effective wavelengths were then applied to the PLSR, RF, and SVR regression models. Similarly, the 10-fold crossvalidation was adopted to calculate the average prediction result of each model. Table 4 presented the optimal prediction results of each spectral selection algorithm in the two adulteration cases.

In the experiments of sesame oil adulterated with soybean oil, the numbers of effective wavelengths extracted by four effective spectral selection algorithms were 83 (PCA), 97 (SPA), 90 (VIP), and 94 (CARS), as seen in Table S1. The PLSR and RF were the optimal methods for the prediction of the adulteration rate of sesame oil, while the SVR did not achieve the optimal performance in any of the experiments with four spectral selection algorithms. The best performance was achieved by the model of PLSR with MSC pretreatment using the effective wavelengths screened by CARS. The $R_{P_{cv}}^{2}$ and $RMSE_{P_{cv}}$ were 0.99656 and 0.01832, respectively. Figure 4c showed the scatter plots and boxplots of the prediction results in the best model (PLSR + MSC + CARS) for sesame oil adulterated with soybean oil. One of the important features was that the outliers in effective wavelength experiment were much less than in the full wavelength experiment (as shown in Figure 4a,c). It performed a slight improvement to the best model of RF with MSC using full wavelengths ($R_{P_{cv}}^{2}$ = 0.99567 and $RMSE_{P_{cv}}$ = 0.01976). This represented that CARS was useful for removing interference and redundant information from the spectral data. In the experiments of rapeseed oil adulterated with soybean oil, the numbers of effective wavelengths were 98 (PCA), 81 (SPA), 90 (VIP), and 144 (CARS), as seen in Table S2. The RF achieved the optimal performance with three effective wavelength selection algorithms. While the PLSR combined with MSC, the pretreatment achieved the best prediction accuracy ($R_{P_{cv}}^{2}$ = 0.99675 and $RMSE_{P_{cv}}$ = 0.01685) using the effective wavelengths screened by CARS, which was also better than the best model of RF with MSC pretreatment using full wavelengths ($R_{P_{cv}}^{2}$ = 0.9929 and $RMSE_{P_{cv}}$ = 0.02504). More concretely, it was consistent with the best prediction model for sesame oil adulterated with soybean oil. Figure 4d showed the detail information of the prediction results in the best prediction model (PLSR + MSC + CARS) of rapeseed oil adulterated with soybean oil.

On the whole, the experiment results demonstrated that the effective wavelengths were beneficial for accurately predicting the adulteration rates of edible oils. The model of PLSR with MSC pretreatment was the best model which achieved the highest accuracy with CARS algorithm in both cases of sesame oil adulteration and rapeseed oil adulteration. It showed good versatility and robustness. The triple pattern (PLSR + MSC + CARS) was the recommended model for predicting the adulteration rates of sesame oil and rapeseed oil. Based on the extracted effective wavelengths, the complexity of prediction model was significantly reduced with the very limited effective wavelengths. In general, the experiment results demonstrated the feasibility and effectiveness of the CARS effective wavelength selection algorithm and highlighted the necessity of eliminating redundant data of the spectra. Therefore, we further investigated the distribution and characteristics of the screened effective wavelengths.

### 3.5. Analysis of the Distribution and Characteristics of the Screened Effective Wavelengths

**Figure 5:**
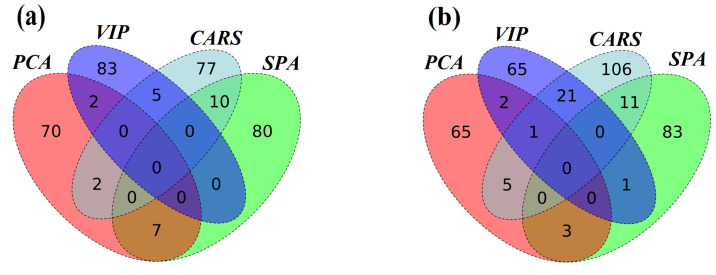
The intersections of the effective wavelengths from the PCA, SPA, VIP, and CARS effective spectral selection algorithms. (**a**) The intersections of the effective wavelengths for the optimal models in sesame oil adulterated with soybean oil. (**b**) The intersections of the effective wavelengths for the optimal models in rapeseed oil adulterated with soybean oil.

The overlap of the effective wavelengths is an important indicator to compare different spectral selection algorithms. In the experiments, the numbers of the effective wavelengths extracted by the four spectral selection algorithms were approximate equilibrium, as shown in Table 4. However, the intersection of the four groups of effective wavelengths was very rare in both of the adulteration cases, as shown in Figure 5. The Venn diagrams represent the exact number of intersections between the pairs of the effective spectral selection algorithms. The patterns of the two adulteration cases were very consistent. The effective wavelengths extracted by different spectral selection algorithms were significantly different. The effective wavelengths based on PCA had almost no overlap with other algorithms. The CARS had very few intersections with VIP and SPA. It indicated that each spectral selection algorithm had its own specific characteristics. Therefore, it should be careful to select the effective spectral selection algorithm for improving the accuracy of the prediction model in edible oil adulteration analysis.

**Figure 6:**
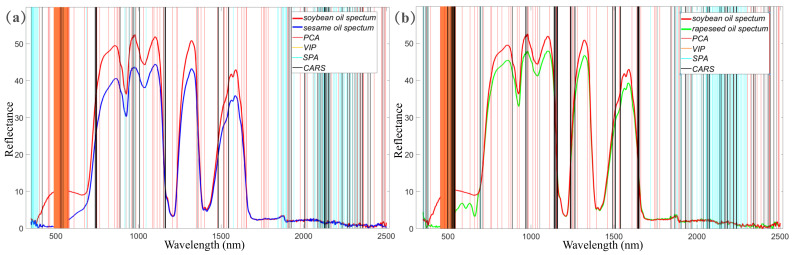
Effective wavelengths selected by PCA, SPA, VIP, and CARS for the best prediction model (PLSR + MSC + CARS). (**a**) Effective wavelengths in sesame oil adulterated with soybean, (**b**) Effective wavelengths in rapeseed oil adulterated with soybean. The red vertical line corresponds to the effective wavelengths selected by SPA; the green vertical line corresponds to the effective wavelengths selected by VIP; and the black vertical line corresponds to the effective wavelengths selected by PCA. The spectrum of rapeseed oil was chosen as the background reference.

Then, the distribution of the selected effective wavelengths in the whole spectra was analyzed, as shown in Figure 6. In general, there was a similar pattern in the case of sesame oil adulterated with soybean oil (Figure 6a) and in the case of rapeseed oil adulterated with soybean oil (Figure 6b). PCA picked out a series of wavelengths spread over the whole band. The PCA identified the effective wavelengths with the larger values in loading vectors which represented that the selected wavelengths contributed to the variance of the original spectral data. However, the wavelengths with large variance of reflectivity may not correlate with adulteration rates. VIP only selected the highly consistent continuous wavelengths in a visible band in two kinds of adulteration experiments. The prediction results were not stable, based on the effective wavelengths screened by VIP, which indicated that the only visible band was not competent to obtain the high-quality prediction results. Obviously, the small piece of continuous wavelengths in visible band cannot represent the most characteristic information of the whole spectrum. Therefore, the VIP is not recommended for selecting effective wavelengths of Vis-NIR spectrum of edible oils. The SPA was inclined to select the wavelengths at the both ends of the Vis-NIR spectrum, which ignored the information around the important peaks and troughs in NIR band. Most of the effective wavelengths screened by SPA were in the range of 1800–2300 nm. The tiny oscillations in the range of 1700–2500 nm (as shown in Figure 3a) could introduce lots of noise to the prediction model. By contrast, CARS screened the effective wavelengths at some discrete positions in the whole spectrum. For example, some effective wavelengths screened by CARS were located at the steepness of the Vis-NIR waveform. The wavelengths at the steepness of the Vis-NIR waveform could be critical features reflecting the adulteration rate of edible oil.

**Figure 7:**
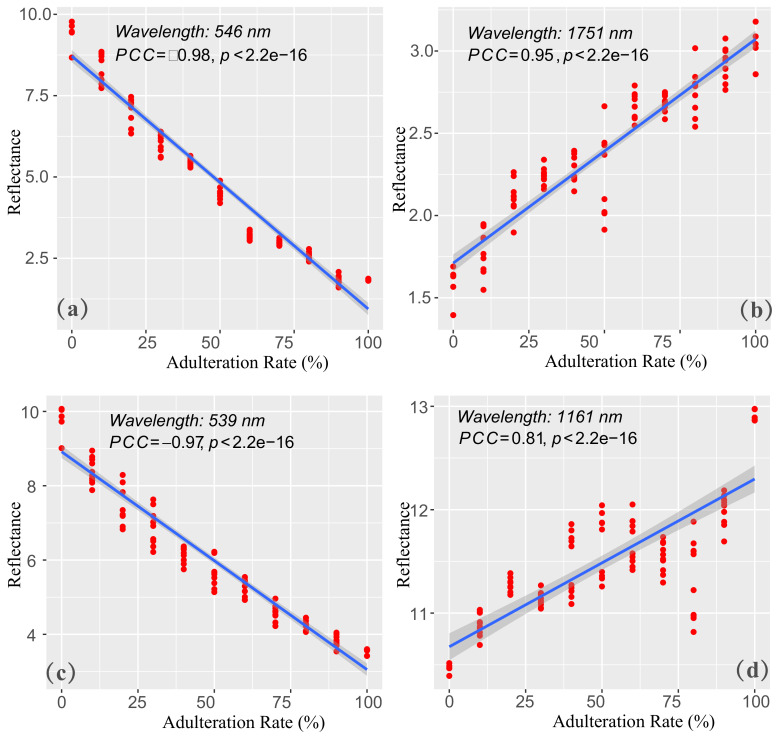
The correlation analysis of the adulteration rates and the reflectance of individual wavelength. The scatter plot of the adulteration rates and the reflectance at the wavelength 546 nm (**a**) and 1751 nm (**b**) in sesame oil adulterated with soybean oil, respectively. The scatter plot of the adulteration rates and the reflectance at the wavelength 539 nm (**c**) and 1161 nm (**d**) in rapeseed oil adulterated with soybean oil, respectively. (The PCC and the *p*-value of the corresponding significance testing were provided in each of the figures. The red dots are the oil samples, and the blue line is the fitting line of data points in each of the figures).

In order to investigate the contribution of individual effective wavelength on adulteration rate, Pearson correlation coefficient (PCC) was employed for evaluating the association relationship between the adulteration rate and the reflectivity in individual wavelength. Based on the best model of the sesame oil adulterated with soybean oil, two representative effective wavelengths (546 and 1751 nm in visible and NIR bands, respectively) were selected for analysis. Figure 7a showed that the reflectivity was significantly correlated with the adulteration rate at a wavelength of 546 nm in the visible band (PCC = −0.98), while there was a significant positive correlation between reflectivity and adulteration rate at wavelength of 1751 nm in NIR band (PCC = 0.95), as shown in Figure 7b. In the experiments of rapeseed oil adulterated with soybean oil, the two representative effective wavelengths (539 and 1161 nm screened by CARS) were chosen from the best prediction model. Figure 7c reflected that the reflectivity were significantly negative correlated with adulteration rates at the wavelength of 539 nm in visible band (PCC = −0.97), and Figure 7d reflected that there was a positive correlation between the reflectivity and the adulteration rates at the wavelength of 1161 nm in NIR band (PCC = −0.81). It was consistent with the experiment of sesame oil adulterated with soybean oil. The results indicated that the soybean oil had strong reflectivity in the visible band. Actually, the sesame oil and rapeseed oil showed darker physical colors than soybean oil in the visible band. Nevertheless, the sesame oil and rapeseed oil performed a strong reflection effect in the NIR band. The analysis demonstrated that CARS was reliable to select the effective wavelengths in the Vis-NIR reflectance spectrum.

## 4. Conclusions

This study evaluated the reliability and feasibility of Vis-NIR reflectance spectroscopy for the rapid and nondestructive discrimination of edible oil adulteration. A Vis-NIR spectroradiometer system was constructed to obtain the reflectance spectra of the sesame oil adulterated with soybean oil and the rapeseed oil adulterated with soybean oil. Four commonly used methods (SNV, MSC, SG smoothing, and WT) were applied to evaluate the impact of pretreatments on spectra data. In the qualitative detection experiments, the accuracy in the prediction set were 100% with the SNV and MSC pretreatments, which suggested that the SVM was a powerful model for oil adulteration type identification. In the quantitative prediction experiments of sesame oil adulterated with soybean oil and rapeseed oil adulterated with soybean oil, the RF method with MSC pretreatment achieved the best prediction effect on the two groups experiments with full wavelengths. Then, the PCA, VIP, SPA and CARS methods were applied to extract the characteristic spectra from the Vis-NIR reflectance spectra. Compared with the prediction performance of full wavelengths, the prediction performance of effective wavelength was slightly improved. According to our results, the model of the triple pattern (PLSR + MSC + CARS) was the recommended method for predicting the adulteration rate of sesame oil adulterated with soybean oil and rapeseed oil adulterated with soybean oil. The Vis-NIR spectra of different edible oils have significantly different amplitudes at a series of characteristic wavelengths. Therefore, the presented methodology can be applied to the adulteration discrimination of other kinds of oils. According to the results reported in this paper, a practical and efficient detection system could be constructed for the fast discrimination of edible oil adulteration in a wide range of application scenarios. To achieve this goal, a simplified reflectance measuring instrument can be designed based on the effective wavelengths. Then, the Vis-NIR spectra data of different edible oils should be measured to retrain the presented model for the nondestructive discrimination of various edible oils adulteration. The enhancement of these related technologies is helpful for the popularization and application of the Vis-NIR spectroradiometer system for oil adulteration detection.

## Figures and Tables

**Table 1:** The prediction accuracy of oil adulteration types in different models.

Model	Pretreatment	${\mathrm{ACC}}_{C_{\mathrm{cv}}}$	${\mathrm{ACC}}_{P_{\mathrm{cv}}}$
SVM	RAW	1.00000	0.97895
	SNV	1.00000	1.00000
	MSC	1.00000	1.00000
	SG	1.00000	0.98947
	WT	1.00000	0.99474
RF	RAW	1.00000	0.97368
	SNV	1.00000	0.99474
	MSC	1.00000	0.99474
	SG	1.00000	0.99474
	WT	1.00000	0.99474
KNN	RAW	0.95556	0.81579
	SNV	0.99766	0.98947
	MSC	0.99766	0.98947
	SG	0.84971	0.66316
	WT	0.96491	0.84737

**Table 2:** Prediction results of sesame oil adulterated with soybean oil using full wavelengths.

Model	Pretreatment	$R_{C_{\mathrm{cv}}}^{2}$	${\mathrm{RMSE}}_{C_{\mathrm{cv}}}$	$R_{P_{\mathrm{cv}}}^{2}$	${\mathrm{RMSE}}_{P_{\mathrm{cv}}}$
PLSR	RAW	0.99945	0.01058	0.96727	0.05285
	SNV	0.99923	0.01201	0.97667	0.04597
	MSC	0.99919	0.01212	0.97721	0.04560
	SG	0.99999	0.00144	0.97126	0.05197
	WT	1.00000	0.00067	0.97282	0.05116
RF	RAW	0.99879	0.01099	0.99201	0.02673
	SNV	0.99921	0.00885	0.99531	0.02030
	MSC	0.99922	0.00882	0.99567	0.01976
	SG	0.99540	0.02142	0.97058	0.05306
	WT	0.99807	0.01388	0.98824	0.03380
SVR	RAW	0.99274	0.02694	0.96790	0.05265
	SNV	0.99330	0.02588	0.98178	0.04114
	MSC	0.99331	0.02585	0.98209	0.04083
	SG	0.99268	0.02706	0.96829	0.05473
	WT	0.99193	0.02840	0.96695	0.05647

**Table 3:** Prediction results of rapeseed oil adulterated with soybean oil using full wavelengths.

Model	Pretreatment	$R_{C_{\mathrm{cv}}}^{2}$	${\mathrm{RMSE}}_{C_{\mathrm{cv}}}$	$R_{P_{\mathrm{cv}}}^{2}$	${\mathrm{RMSE}}_{P_{\mathrm{cv}}}$
PLSR	RAW	0.99965	0.00873	0.98420	0.03775
	SNV	0.99955	0.00948	0.99019	0.02902
	MSC	0.99953	0.00958	0.99008	0.02919
	SG	1.00000	0.00076	0.98258	0.04026
	WT	1.00000	0.00036	0.98210	0.04097
RF	RAW	0.99896	0.01018	0.99353	0.02454
	SNV	0.99891	0.01038	0.99277	0.02533
	MSC	0.99894	0.01024	0.9929	0.02504
	SG	0.99871	0.01133	0.99124	0.02862
	WT	0.99890	0.01044	0.99390	0.02372
SVR	RAW	0.99235	0.02766	0.97932	0.04443
	SNV	0.99415	0.02418	0.98820	0.03281
	MSC	0.99410	0.02429	0.98814	0.03293
	SG	0.99289	0.02667	0.97625	0.04716
	WT	0.99237	0.02763	0.97201	0.05162

**Table 4:** Prediction results obtained by the better model using effective wavelengths.

Adulteration Type	Method	Pretreatment	Spectral Selection	Number	$R_{C_{\mathrm{cv}}}^{2}$	${\mathrm{RMSE}}_{C_{\mathrm{cv}}}$	$R_{P_{\mathrm{cv}}}^{2}$	${\mathrm{RMSE}}_{P_{\mathrm{cv}}}$
Sesame oil adulterated with soybean oil	RF	SNV	PCA	83	0.99881	0.01091	0.99275	0.02470
	PLSR	SNV	SPA	97	0.99621	0.02107	0.94951	0.06516
	RF	SNV	VIP	90	0.99896	0.01014	0.99377	0.02364
	PLSR	MSC	CARS	94	0.99911	0.01022	0.99656	0.01832
Rapeseed oil adulterated with soybean oil	RF	RAW	PCA	98	0.99836	0.01280	0.99047	0.03050
	RF	RAW	SPA	81	0.98602	0.03730	0.92778	0.08287
	RF	RAW	VIP	90	0.99938	0.00789	0.99587	0.01913
	PLSR	MSC	CARS	144	0.99914	0.00991	0.99675	0.01685

## Data Availability

Not applicable.

## Supplementary Materials

The following are available at https://www.mdpi.com/article/10.3390/bios11120492/s1, Table S1: The effective wavelengths screened by CARS in adulteration experiment of sesame oil and soybean oil, Table S2: The effective wavelengths screened by CARS in adulteration experiment of rapeseed oil and soybean oil.
