# Supplementary material for: Reflectance Spectroscopy with Multivariate Methods for Non-Destructive Discrimination of Edible Oil Adulteration
Source: Biosensors (Basel). 2021 Dec 2;11(12):492. doi: 10.3390/bios11120492 (PMC8699652; doi:10.3390/bios11120492)
Supplement: Supplementary file 1 [file biosensors-11-00492-s001.zip › biosensors-1438142-supplementary.pdf]

Supplementary material: Reflectance spectroscopy with multivariate methods for non-destructive discrimination of edible oil adulteration

Ning Su [1,2], Shizhuang Weng[3], Liusan Wang [1,2]* and Taosheng Xu[1,2]

[1] Institute of Intelligent Machines, Hefei Institutes of Physical Science, Chinese Academy of Sciences, Hefei 230031, China

[2] Intelligent Agriculture Engineering Laboratory of Anhui Province, China, Hefei 230031, China

[3] National Engineering Research Center for Agro-Ecological Big Data Analysis and Application, Anhui University, Hefei 230601, China

---

Table S1. The effective wavelengths screened by CARS in adulteration experiment of sesame oil and soybean oil.

Method	Number	wavelengths
PCA	83	402, 524, 749, 856, 957, 1085, 1233, 1379, 1535, 1639, 1772,1895, 2064, 2236, 2362, 2491, 353, 464, 610, 766, 870, 971, 1113, 1350, 1600, 1744, 1906, 2021, 2123, 2237, 2346, 2479, 454, 571, 673, 820, 925, 1030, 1150, 1356, 1520, 1626, 1763, 1878, 2062, 2170, 2360, 2470, 353, 458, 578,692, 884, 985, 1094, 1205, 1400, 1516, 1652, 1757, 1863, 1979, 2093, 2210, 2325, 2426, 355, 464, 582, 687, 876, 980, 1130, 1234, 1364, 1479, 1653, 1785, 1903, 2011, 2162, 2308, 2496
SPA	97	2157, 1359, 2133, 972, 1007, 943, 2019, 973, 2141, 2069, 2484, 1881, 2394, 2123, 398, 2284, 2315, 2234, 391, 687, 924, 1574, 1895, 2337, 2469, 974, 2446, 2343, 2146, 2330, 1848, 2323, 2364, 2493, 2388, 1890,2464, 2093, 2413, 2418, 386, 1898, 2296, 2149, 2104, 1873, 2180, 2397, 2454, 2164, 2261, 1878, 2306, 2422, 380, 1841, 2403, 2226, 2474, 2431, 2425, 2409, 370, 2246, 2064, 2056, 982, 2120, 2195, 2096, 2355, 2117, 2185, 358, 1868, 1046, 2218, 355, 2371, 2488, 2435, 375, 2136, 366, 361, 916, 1941, 2221, 363, 2200, 2380, 2083, 350, 2500, 2239, 2268, 2350
VIP	90	530, 525, 524, 531, 529, 526, 523, 532, 528, 527, 522, 533, 534, 521, 535, 520, 517, 518, 519, 538, 537, 536, 539, 516, 540, 541, 515, 543, 542, 544, 514, 545, 513, 546, 512, 547, 511, 510, 548, 549, 509, 550, 551, 508, 507, 552, 553, 554, 506, 555, 556, 505, 557, 504, 558, 503, 559, 502, 560, 501, 561, 562, 500, 563, 499, 564, 498, 565, 497, 566, 567, 496, 568, 495, 569, 494, 570, 493, 571, 572, 492, 573, 491, 574, 575, 490, 576, 489, 577, 488
CARS	94	353, 417, 418, 530, 531, 532, 538, 546, 737, 738, 740, 742, 743, 744, 745, 746, 747, 888, 890, 944, 965, 966, 972, 1006, 1007, 1008, 1153, 1160, 1161, 1162, 1163, 1164, 1165, 1481, 1482, 1483, 1484, 1515, 1544, 1545, 1546, 1547, 1907, 1916, 1917, 1926, 1927, 2005, 2006, 2007, 2082, 2083, 2091, 2099, 2100, 2109, 2114, 2124, 2125, 2126, 2127, 2128, 2131, 2132, 2136, 2137, 2141, 2148, 2149, 2152, 2153, 2154, 2229, 2236, 2266, 2267, 2278, 2279, 2280, 2297, 2316, 2323, 2338, 2344, 2345, 2354, 2355, 2356, 2387, 2394, 2403, 2404, 2475, 2486

Table S2. The effective wavelengths screened by CARS in adulteration experiment of rapeseed oil and soybean oil.

Method	Number	wavelengths
PCA	98	396, 506, 661, 763, 867, 1011, 1113, 1249, 1355, 1535, 1744, 1848, 1961, 2064, 2201, 2385, 2488, 363, 530, 633, 758, 917, 1026, 1147, 1254, 1355, 1498, 1608, 1759, 1872, 2026, 2219, 2323, 2438, 354, 481, 585, 715, 879, 980, 1152, 1355, 1531, 1647, 1752, 1890, 2064, 2170, 2304, 2436, 352, 454, 585, 698, 799, 985, 1153, 1279, 1394, 1661, 1865, 2064, 2195, 2361, 2494, 369, 546, 678, 825, 929, 1039, 1235, 1339, 1461, 1637, 1776, 1896, 2062, 2238, 2342, 2489
SPA	81	507, 1761, 1999, 1866, 2389, 2189, 2393, 2378, 2415, 2203, 2303, 2448, 2149, 2216, 2263, 2143, 2059, 379, 2252, 362, 2018, 1881, 2126, 2056, 2072, 2053, 2400, 375, 2269, 2090, 2373, 2345, 2316, 2488, 2340, 2180, 2077, 2112, 2479, 2040, 2185, 2242, 2473, 2233, 2228, 2286, 2350, 2105, 1974, 2332, 2277, 1842, 2171, 2146, 2247, 2442, 2097, 2153, 2132, 1862, 2163, 2027, 2194, 2499, 2198, 696, 2433, 2325, 2292, 2123, 2064, 2037, 1905, 2320, 1719, 1898, 352, 2468, 2298, 2367, 2408, 367, 2423, 1648, 2115, 2094, 2208, 1957, 1874, 2458, 2310, 2212, 2139, 2136, 2157, 2220, 2080, 2358
VIP	90	516, 515, 517, 514, 513, 512, 518, 511, 519, 510, 520, 509, 521, 508, 522, 507, 523, 506, 524, 525, 505, 526, 504, 527, 503, 502, 534, 532, 535, 528, 501, 533, 536, 531, 529, 530, 537, 500, 499, 538, 498, 497, 496, 495, 494, 490, 491, 493, 489, 488, 486, 487, 492, 485, 539, 484, 482, 481, 483, 480, 477, 478, 479, 540, 476, 475, 474, 541, 473, 472, 470, 471, 469, 468, 542, 464, 467, 465, 466, 463, 462, 543, 459, 461, 460, 458, 457,456, 455, 544
CARS	144	350, 354, 368, 374, 375, 381, 497, 498, 499, 500, 517, 521,522, 524, 525, 527, 528, 530, 531, 532, 535, 536, 537, 538, 539, 540, 541, 542, 545, 692, 695,698, 708, 887, 889, 966, 968, 970, 972, 979, 1053, 1138, 1139, 1140, 1141, 1142, 1143, 1147,1148, 1149, 1155, 1156, 1157, 1158, 1159, 1160, 1161, 1237, 1238, 1239, 1240, 1241, 1242, 1263, 1264, 1265, 1266, 1490, 1491, 1492, 1502, 1507, 1508, 1509, 1510, 1537, 1538, 1539, 1540, 1641, 1642, 1643, 1644, 1645, 1646, 1648, 1649, 1650, 1651, 1652, 1842, 1843, 1865, 1866, 1903, 1904,1929, 1930, 1931, 1939, 1946, 1968, 1969, 2036, 2037, 2060, 2061, 2069, 2074, 2075, 2076, 2137,2138, 2145, 2146, 2164, 2165, 2172, 2180, 2181, 2182, 2183, 2220, 2221, 2222, 2236, 2237, 2238, 2239, 2329, 2340, 2341, 2349, 2350, 2401, 2408, 2422, 2431, 2432, 2440, 2441, 2462, 2463, 2477
